# Construction and validation of a predictive model for meningoencephalitis in pediatric scrub typhus based on machine learning algorithms

**DOI:** 10.1080/22221751.2025.2469651

**Published:** 2025-02-18

**Authors:** Yonghan Luo, Wenrui Ding, Xiaotao Yang, Houxi Bai, Feng Jiao, Yan Guo, Ting Zhang, Xiu Zou, Yanchun Wang

**Affiliations:** a Second Department of Infectious Disease, Kunming Children's Hospital, Kunming, People’s Republic of China; b Yunnan Key Specialty of Pediatric Infection (Training and Education Program)/Kunming Key Specialty of Pediatric Infection, Kunming, People's Republic of China; c Faculty of Life Science and Technology, Kunming University of Science and Technology, Kunming, People’s Republic of China

**Keywords:** Scrub typhus, pediatrics, meningoencephalitis, machine learning, predictive model

## Abstract

To retrospectively analyze the clinical characteristics of pediatric scrub typhus (ST) with meningoencephalitis (STME) and to construct and validate predictive models using machine learning.

Clinical data were collected from 100 cases of pediatric STME and matched with data from 100 ST cases without meningitis using propensity-score matching. Risk factors for STME in pediatrics were identified through the least absolute shrinkage and selection operator (LASSO) regression analysis. Six predictive models—Logistic Regression, K-Nearest Neighbors, Naive Bayes, Multi-layer Perceptron(MLP), Random Forest, and XGBoost—were constructed using the training set and evaluated for performance, with validation conducted on the test set. The Shapley Additive Explanations (SHAP) method was applied to rank the importance of each variable.

All children improved and were discharged following treatment with azithromycin/doxycycline (1/99). Twelve variable features were identified through the LASSO regression. Of the six predictive models developed, the XGBoost model demonstrated the highest performance in the training set (AUC = 0.926), though its performance in the test set was moderate (AUC = 0.740). The MLP model exhibited robust predictive performance in both training and test sets, with AUCs of 0.897 and 0.817, respectively. Clinical decision curve analysis indicated that the MLP and XGBoost models provide significant clinical utility. SHAP analysis identified the most important predictors for STME as ferritin, white blood cell count, edema, prothrombin time, fibrinogen, duration of pre-admission fever, eschar, activated partial thromboplastin time, splenomegaly, and headache. The MLP and XGBoost models showed strong predictive capability for pediatric STME, with favorable outcomes following doxycycline-based therapy.

## Introduction

Scrub typhus (ST) is a natural focal disease transmitted by bites of larval mites and caused by *Orientia tsutsugamushi* [1]. Clinically, it is characterized by high fever, eschar or ulcer at the bite site, rash, lymphadenopathy, and hepatosplenomegaly [2]. In the second week of illness, multiple organ damage often occurs, with primary manifestations including pneumonia, myocarditis, liver dysfunction, and encephalitis. Reports indicate that 20%–25% of hospitalized patients with ST experience neurological complications, which are associated with high mortality [3,4], and the case fatality rate in pediatric cohorts is 6.1% [5]. The exact pathogenesis of neurological involvement in ST remains unclear. Proposed mechanisms include direct invasion by the pathogen, a vasculitis-like process, or immune-mediated damage [6,7]. *Orientia tsutsugamushi* infiltrates the central nervous system (CNS) by invading vascular endothelial cells, releasing cytokines that compromise endothelial integrity, causing fluid leakage. This process triggers localized platelet aggregation and proliferation of multinucleated and mononuclear macrophages, leading to focal occlusive vasculitis and microthrombi formation near the meninges, brain parenchyma, and venous sinuses [8,9]. Studies in pediatric CNS infection surveillance in Cambodia, Vietnam, Laos, Myanmar, and Thailand report that rickettsial infections account for 1%–18% of CNS infections in children [10–14]. When ST affects the CNS, meningitis is the most common manifestation, while rarer clinical presentations include cranial nerve palsies, plexopathy, transverse myelitis, neuroleptic malignant syndrome, and Guillain-Barré syndrome [7,15].

Early diagnosis is crucial for successful treatment [7]. scrub typhus with meningoencephalitis (STME) patients may present with symptoms such as headache, vomiting, consciousness disturbances, sensory impairment, and neck stiffness. However, fever is a more common manifestation of STME in children, which increases the risk of misdiagnosis. Most studies on acute encephalitic syndrome associated with STME have focused on adults, with limited research in pediatric populations. Some retrospective studies [16] have identified eschar, capillary leakage, hepatomegaly, and splenomegaly as risk factors for STME. However, the clinical utility of using single-variable risk factors is limited. In recent years, machine learning models, with their strengths in big data analysis, artificial intelligence, and visualization, have been widely applied in disease prediction [17,18]. Nevertheless, there is no reported machine learning model for STME in children. Therefore, we reviewed ten years of STME clinical data from our hospital and developed and validated various machine learning models to inform early diagnosis and treatment of STME patients.

## Materials and methods

### Study population

**Figure 1.**
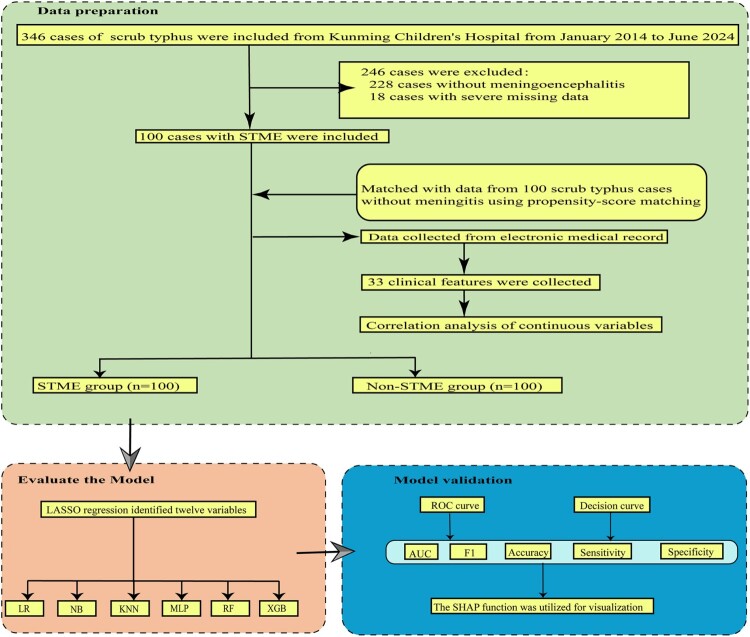
The flowchart of the study.

Clinical data were collected for 100 cases of pediatric STME, admitted between January 2014 and June 2024. Using propensity-score matching, these cases were compared with clinical data from 100 children with ST but without encephalitis (The flowchart of this study is shown in Figure 1). This study received approval from the Ethics Committee at Kunming Children's Hospital (2023-03-320-K01).

Inclusion criteria:

1. Patients younger than 18 years who were hospitalized.
2. Diagnosis in accordance with the 5th edition Pediatric Infectious Diseases diagnostic guidelines for ST [19]. Clinical diagnosis of ST was made for patients who:
  - Had a recent history (within three weeks) of outdoor exposure in endemic regions, presenting with an acute high fever accompanied by typical symptoms such as an eschar or ulcer, rash, swollen lymph nodes, and other characteristic clinical signs.
  - Showed at least one positive laboratory result, which could include specific IgM antibodies, a fourfold rise in serum antibody levels (detected through the Weil-Felix test), or pathogen identification using polymerase chain reaction (PCR).
  - Were highly suspected of having ST, even if a definitive diagnosis was unconfirmed, and experienced fever resolution within 48 h after starting treatment with either azithromycin or doxycycline.
3. Comprehensive lumbar puncture examination, revealing no evidence of bacterial infection in cerebrospinal fluid culture.

Exclusion criteria:

1. Presence of pre-existing conditions, such as congenital heart disease, hematological malignancies, or immune deficiencies.
2. Substantial gaps in clinical information.

For patients with a disease course marked by fever lasting more than one week or presenting neurological symptoms such as vomiting, headache, or lethargy suggestive of central nervous system infection, a lumbar puncture examination was performed. Presence of one or more of the following findings—CSF pleocytosis, meningeal enhancement, or parenchymal inflammation on contrast-enhanced CT or MRI of the brain—qualifies classification into the STME group [20]. Additionally, 100 ST patients without meningitis were designated as the control (non-STME) group.

### Study variables and data extraction

Gather information on ST patients from the hospital's medical record system, covering demographics, clinical manifestations, laboratory findings, treatment methods, and prognosis.

### Predictive model construction and evaluation

Least Absolute Shrinkage and Selection Operator (LASSO) regression analysis was performed using R software (version 4.4.1) to identify factors influencing the occurrence of STME. Six predictive models were developed: Logistic Regression, K-Nearest Neighbors (KNN), Naive Bayes, Multilayer Perceptron (MLP), Random Forest, and Extreme Gradient Boosting (XGBoost) [21–23]. Logistic Regression, a well-established and interpretable algorithm for binary classification problems, was used as a baseline model for comparison. KNN, a non-parametric method, was included for its effectiveness in smaller datasets and its ability to capture complex, non-linear relationships. Naive Bayes was selected for its computational efficiency and strong performance in scenarios where feature independence assumptions approximately hold. MLP, a neural network model, was utilized to explore non-linear interactions between features, making it suitable for datasets with complex relationships or latent patterns that are not explicitly apparent.

Random Forest, an ensemble-based algorithm, was chosen for its robustness against overfitting, its ability to handle missing data, and its capability to provide interpretable feature importance rankings. XGBoost, a popular gradient boosting algorithm, was included due to its superior predictive performance, computational speed, and effectiveness in handling imbalanced datasets. Model performance was evaluated based on the area under the roc curve (AUC) and decision curve analysis (DCA) to assess both discriminative ability and clinical utility. The shapley additive explanations (SHAP) analysis was used to interpret model results and assess feature importance.

### Statistical methods

Analysis was conducted using R software. Variables with a missing rate >20% and outliers were excluded, and missing data were imputed using multiple imputation methods. For continuous variables following a normal distribution, data were presented as mean ± standard deviation (x ± s) and assessed using the t-test. Data not following a normal distribution were presented as M (P25, P75) and analyzed using the Mann-Whitney U test. Categorical data were presented as rates [n (%)] and evaluated accordingly. A significance level of *P* < 0.05 indicated statistical significance.

## Results

### Comparison of baseline characteristics between STME group and non-STME group

In this study, each group—STME and Non-STME—consisted of 100 patients. The median age of the STME group was significantly younger than that of the non-STME group (3.8 vs. 5.11 years, *P* = 0.046). There was no significant difference in sex distribution between the groups (*P* = 1.00), with males representing 48% of the total sample. Variables such as duration of pre-hospital fever, eschar, edema, WBC, PT, and ferritin demonstrated statistically significant differences between the groups (*p* < 0.05), while other variables showed no significant differences **(see** Table 1).

**Table 1.** Comparison of clinical characteristics between the non-STME group and STME group in pediatric scrub typhus.

	Total (*n* = 200)	Non-STME (*n* = 100)	STME (*n* = 100)	*p*
**Patient characteristics**				
Sex (male) [*n* (%)]				1
No	105 (52)	52 (52)	53 (53)	
Yes	95 (48)	48 (48)	47 (47)	
Age (median [IQR]), years	4.6 (2.6, 8)	5.11 (2.9, 8.83)	3.8 (2.5, 7.32)	0.046
History of field exposure [n (%)]				0.886
No	84 (42)	43 (43)	41 (41)	
Yes	116 (58)	57 (57)	59 (59)	
Length of stay (median [IQR]), d	7 (6, 9)	7 (6, 8)	7 (6, 9)	0.172
The duration of Pre-hospital fever (median [IQR]), days	8 (6, 10)	7 (5, 10)	8 (6.75, 10)	0.043
**Symptoms**				
Fever [n (%)]				0.17
No	9 (4)	2 (2)	7 (7)	
Yes	191 (96)	98 (98)	93 (93)	
Cough [n (%)]				0.322
No	96 (48)	52 (52)	44 (44)	
Yes	104 (52)	48 (48)	56 (56)	
Headache [n (%)]				0.498
No	155 (78)	80 (80)	75 (75)	
Yes	45 (22)	20 (20)	25 (25)	
Vomiting [n (%)]				0.357
No	164 (82)	85 (85)	79 (79)	
Yes	36 (18)	15 (15)	21 (21)	
Abdominal pain [n (%)]				0.739
No	153 (76)	75 (75)	78 (78)	
Yes	47 (24)	25 (25)	22 (22)	
**Physical signs**				
Eschar [n (%)]				<0.001
No	52 (26)	38 (38)	14 (14)	
Yes	148 (74)	62 (62)	86 (86)	
Edema [n (%)]				<0.001
No	140 (70)	82 (82)	58 (58)	
Yes	60 (30)	18 (18)	42 (42)	
Lymphadenopathy [n (%)]				0.671
No	98 (49)	51 (51)	47 (47)	
Yes	102 (51)	49 (49)	53 (53)	
Hepatomegaly [n (%)]				0.089
No	107 (54)	60 (60)	47 (47)	
Yes	93 (46)	40 (40)	53 (53)	
Splenomegaly [n (%)]				0.057
No	126 (63)	70 (70)	56 (56)	
Yes	74 (37)	30 (30)	44 (44)	
**Laboratory Tests (peripheral blood)**				
WBC (median [IQR]), × 10^9^/L	8.84 (5.43, 11.74)	7.59 (4.58, 9.8)	9.64 (7.29, 13.28)	<0.001
PLT (median [IQR]), × 10^9^/L	107 (56.75, 171)	110 (64, 176)	103.5 (42.75, 162)	0.346
Hemoglobin (Mean ± SD), g/L	111.28 ± 16.19	112.34 ± 15.69	110.23 ± 16.68	0.358
Eosinophils (median [IQR]), × 10^9^/L	0.01 (0, 0.02)	0 (0, 0.01)	0.01 (0, 0.02)	0.129
ALT (median [IQR]), U/L	64.5 (43, 121.88)	58.5 (41, 97.75)	74.5 (44, 143)	0.12
AST (median [IQR]), U/L	78 (54, 151.12)	78 (54, 129.28)	78 (54.75, 166)	0.455
TBil (median [IQR]), umol/L	8.2 (6.4, 10.85)	8.7 (6.5, 10.57)	8.2 (6.4, 11)	0.815
Albumin (median [IQR]), g/L	30.9 (27.08, 35.23)	31.72 (27.75, 35.82)	30.1 (27.08, 34.47)	0.209
LDH (median [IQR]), U/L	571 (442.5, 729.7)	542.25 (444, 705.25)	589.5 (435.5, 763.35)	0.338
BUN (median [IQR]), mmol/L	3.73 (2.98, 4.82)	3.65 (2.98, 4.73)	3.8 (2.98, 5.02)	0.322
SCr (median [IQR]), umol/L	29.6 (22.87, 38.25)	31 (23, 39.15)	28.05 (21.9, 36.54)	0.19
UA (median [IQR]), umol/L	240.5 (190.38, 295)	248 (187, 291.55)	233 (192.75, 300)	0.923
Fibrinogen (median [IQR]), g/L	2.37 (1.58, 2.8)	2.44 (1.58, 3.23)	2.2 (1.57, 2.62)	0.148
APTT (median [IQR]), s	38.48 (31.7, 42.92)	38.48 (30.3, 42.85)	38.48 (32.27, 42.92)	0.401
PT (median [IQR]), s	13.9 (12.7, 15.74)	14.05 (13, 17.93)	13.5 (12, 15.74)	<0.001
CRP (median [IQR]), mg/L	32.76 (13.06, 63.1)	32.35 (13.91, 50.99)	33.73 (12.43, 74.53)	0.711
PCT (median [IQR]), ng/ml	2.87 (0.88, 4.36)	2.69 (1.06, 4.85)	3.48 (0.82, 3.68)	0.943
Ferritin (median [IQR]), ng/ml	574 (276.8, 912.89)	410 (215, 912.19)	912.19 (428.48, 1056.5)	<0.001

STME (scrub typhus with meningoencephalitis), WBC (white blood cell), PLT (platelet), CRP (C -reactive protein), PCT (procalcitonin), ALT (alanine aminotransferase), AST (alanine aminotransferase), TBil(total bilirubin), SCr(serum creatinine), BUN(blood urea nitrogen), UA (uric acid), CK-MB(creatine kinase isoenzyme), LDH(lactate dehydrogenase), PT(prothrombin time), APTT(activated partial thromboplastin time)

### Characteristics of cerebrospinal fluid parameters in pediatric STME

In the CSF of children with STME, the median WBC count was 29.5 × 10⁶/L, with an interquartile range (IQR) of 18.0–52.8 × 10⁶/L. The monocyte percentage in the CSF was notably high, with a median of 82.7% (IQR 72.4% to 89.3%). The average glucose level in the CSF was 3.20 mmol/L (SD ± 0.77). The median CSF protein level was 0.24 g/L (IQR 0.13–0.40 g/L), and the average CSF chloride concentration was 122.1 mmol/L (SD ± 4.5).

### Selection of variables

**Figure 2.**
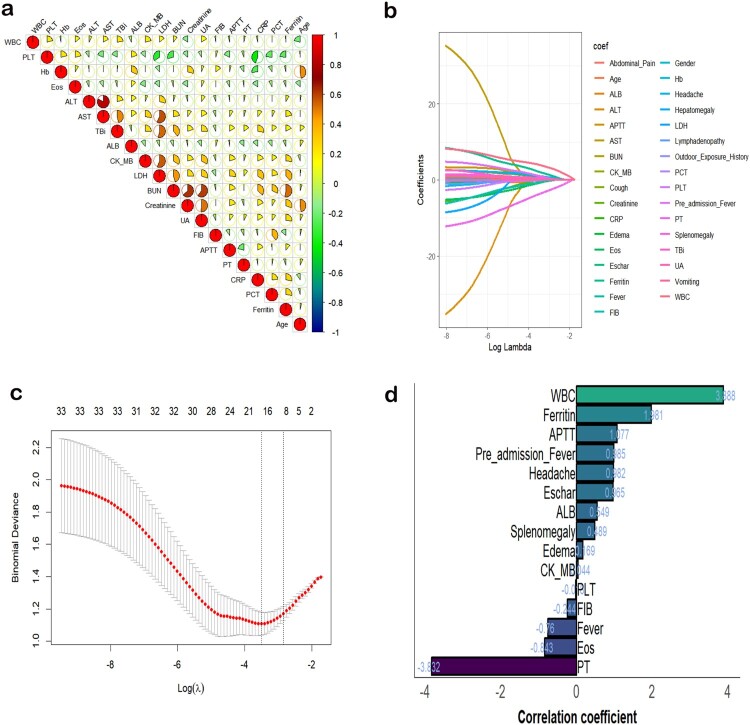
Process of Selecting Variables through LASSO Regression. **(a)** Heatmap of Correlations among Continuous Variables, with larger pie segments indicating stronger correlations. **(b)** LASSO coefficient profiles of the 33 variables. The coefficient profile plot was produced against the log (λ) sequence. The best penalty coefficient lambda was selected using a tenfold cross-validation and minimization criterion. **(c)** By verifying the optimal parameter (λ) in the LASSO model, the binomial deviance curve was plotted versus log (λ) and dotted vertical lines were drawn. 12 variables with nonzero coefficients were selected by lambda.se. **(d)** Bar Chart of Coefficients for Variables with Nonzero Coefficients Selected by Lambda.min.

We initially performed a correlation analysis among the continuous variables, revealing significant correlations, such as ALT and AST (Figure 2(a)). To reduce multicollinearity, we applied LASSO regression for feature selection (Figure 2(b and c)). The predictive variables selected included: “fever,” “headache,” “eschar,” “edema,” “splenomegaly,” “WBC,” “eosinophils,” “fibrinogen,” “APTT,” “PT,” “ferritin,” and “duration of pre-admission fever.” Coefficients for each selected variable are shown in Figure 2(d).

### SHAP analysis and interpretation

**Figure 3.**
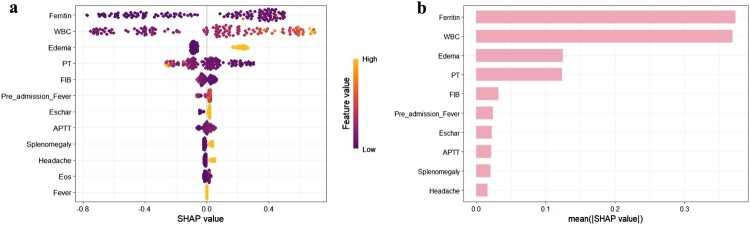
SHAP Interpretation of the Model. **(a).** SHAP values for all samples and features, with each row representing a feature and the x-axis indicating the SHAP value. Yellow dots denote higher feature values, while purple dots indicate lower feature values. **(b).** Ranking of variable importance based on average SHAP values.

**Figure 4.**
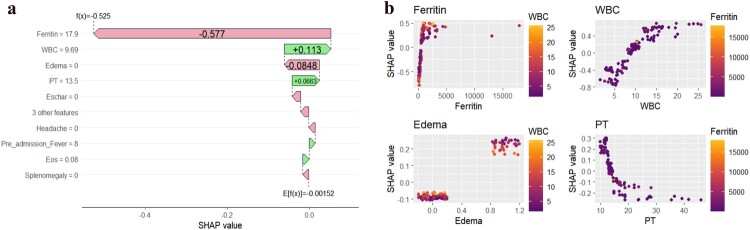
**(a)** SHAP predictions for STME. The pink arrows indicate that the feature exerts a negative influence on the prediction, while the light green arrows signify a positive impact. The arrow length reflects the impact magnitude, with longer arrows signifying a more substantial effect on the prediction. **(b).** Dependency plot illustrating relationships among the four variables with the highest SHAP values.

SHAP visualization in Figure 3(a) ranks the top 10 risk factors for STME, with the highest importance factors being ferritin, WBC, edema, prothrombin time, fibrinogen, pre-admission fever duration, eschar, APTT, splenomegaly, and headache. The intensity of yellow indicates higher risk values, while deeper purple denotes lower risk values. Figure 3(b) displays the SHAP values from highest to lowest for each feature. A representative prediction case for non-STME is provided in Figure 4(a) to illustrate model interpretability. Additionally, partial dependence plots among the four most influential variables are shown in Figure 4(b) to depict the relationships among variables.

### Model construction and validation

**Figure 5.**
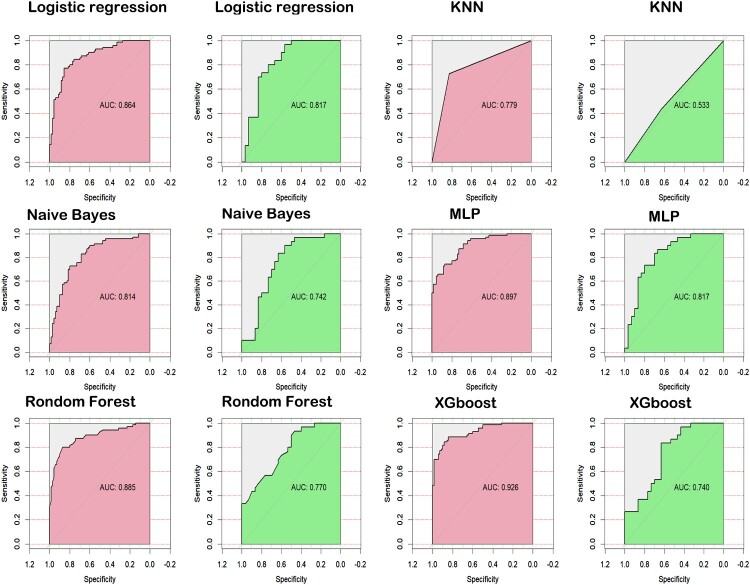
ROC Curves for Six Machine Learning Models. Pink represents the training set, and light green represents the test set.

**Figure 6.**
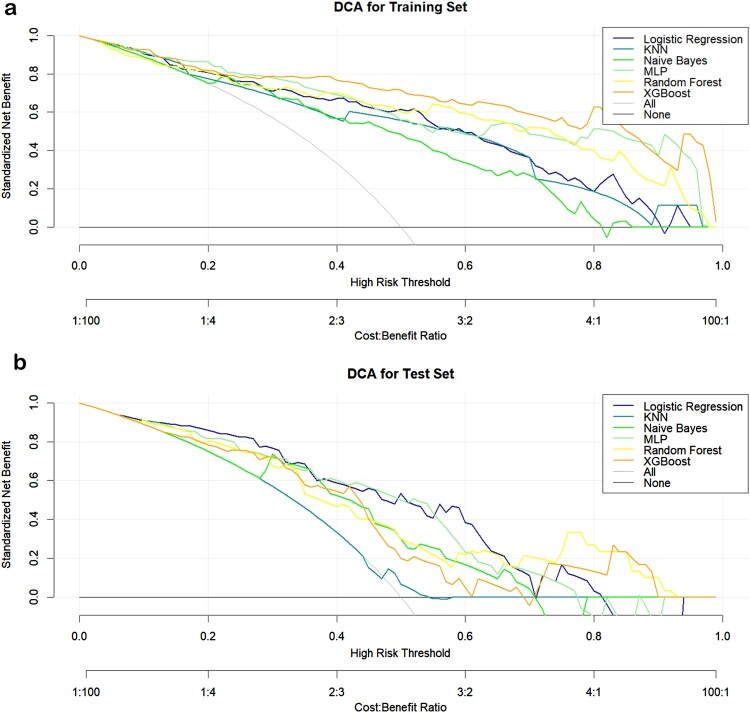
Decision Curves for Six Machine Learning Models: **(a)** Training Set, **(b)** test Set.

All subjects were randomly divided into training and test sets in a 7:3 ratio. Using the 12 variables identified through LASSO regression, six machine learning algorithms—Logistic Regression, KNN, Naive Bayes, MLP, Random Forest, and XGBoost—were employed to construct and validate predictive models for the training set. The XGBoost model exhibited the best predictive performance in the training set with an AUC of 0.926 (95% CI 0.884–0.968). However, its performance in the test set was moderate, with an AUC of 0.740 (95% CI 0.613–0.867). In contrast, the MLP model demonstrated strong predictive performance in both the training and test sets, with AUCs of 0.897 (95% CI 0.848–0.946) and 0.817 (95% CI 0.706–0.927), respectively (**see** Figure 5). A comprehensive performance summary for each model is presented in Table 2. The results of the DCA showed that when the threshold probability was between 0.10 and 0.08, the net benefit rate of the six model in predicting STME was greater than 0 in both the training set and the validation set. And the MLP and XGBoost models provided the highest net benefit across most threshold ranges, suggesting good clinical utility (see Figure 6).

**Table 2.** Performance of six models.

	AUC (95% CI)	F1	Accuracy	Sensitivity	Specificity
Logistic Regression(train)	0.864(0.804–0.924)	0.814	0.807	0.843	0.771
Logistic Regression(test)	0.817(0.704–0.929)	0.746	0.750	0.733	0.767
KNN (train)	0.779(0.710–0.847)	0.789	0.779	0.829	0.729
KNN (test)	0.533(0.408–0.659)	0.576	0.533	0.633	0.433
Naive Bayes(train)	0.814(0.743–0.886)	0.745	0.721	0.814	0.629
Naive Bayes(test)	0.742(0.611–0.874)	0.696	0.650	0.800	0.500
Multi-layer Perceptron (train)	0.897(0.848–0.946)	0.756	0.779	0.686	0.871
Multi-layer Perceptron (test)	0.817(0.706–0.927)	0.741	0.767	0.667	0.867
Random Forest(train)	0.885(0.828–0.941)	0.752	0.779	0.671	0.886
Random Forest(test)	0.770(0.653–0.887)	0.630	0.667	0.567	0.767
XGBoost(train)	0.926(0.884–0.968)	0.857	0.850	0.900	0.800
XGBoost(test)	0.740(0.613–0.867)	0.657	0.617	0.733	0.500

AUC (Area Under the Roc Curve), F1(F1 score), KNN (K-Nearest Neighbors), XGBoost (Extreme Gradient Boosting).

## Discussion

In this study, we conducted a retrospective analysis of the clinical characteristics of STME. Using LASSO regression, we selected risk factors such as ferritin, WBC, edema, and prothrombin time to develop six predictive models. Model validation demonstrated that both the XGBoost and MLP exhibited robust discriminative power and clinical utility. Furthermore, we employed the SHAP function to visualize the predictive models. We hope that our findings will provide valuable insights for the clinical diagnosis and management of STME.

Diagnosing ST generally requires a combination of exposure history, clinical features, and serological tests. Neurological symptoms of ST are often pronounced in adults, manifesting as vomiting and, in severe cases, limb weakness or sensory alterations [24]. However, our study found that in children with STME, fever might be the only presenting symptom, with low incidence of headache or vomiting; prolonged, unexplained fever was a key risk factor for STME. In clinical practice, we have observed that ST cases with fever lasting more than one week are more likely to involve encephalitis, suggesting lumbar puncture and CSF analysis to ascertain CNS infection. In previous years, due to the limited application of advanced diagnostic techniques like next-generation sequencing (NGS), we did not perform *Orientia tsutsugamushi* testing in CSF for patients with significantly elevated CSF cell counts after lumbar puncture. However, with the recent advancements in NGS technology, we have sent CSF samples for NGS analysis in some ST patients with high cell counts. The detection rate of *Orientia tsutsugamushi* in these cases has been relatively low, which suggests that the pathogenesis of STME may more likely involve immune-mediated damage. Nevertheless, this hypothesis warrants further investigation. PCR and NGS offer significant advantages in specificity, accuracy, and early diagnosis, with increasing reports [25,26] of mNGS proving valuable for early diagnosis of rickettsial infections and improving detection rates of rare neurological complications in ST.

Our study identified several predictive factors, including inflammatory and coagulation markers such as ferritin, white blood cell count, fibrinogen, and fever prior to admission. As is well known, prolonged fever (>7 days), decreased white blood cell and platelet counts, fibrinogen <1.5 g/L, and ferritin >500 μg/L are clinical features of hemophagocytic lymphohistiocytosis(HLH). These same predictive factors suggest a potential association between encephalitis and HLH [27]. While our findings indicated that most pediatric STME improved after treatment, HLH can be fatal. Reports [28] indicated that 28% of HLH cases in pediatric intensive care units (PICUs) are secondary to ST, with high mortality rates, underscoring the necessity of early encephalitis detection and treatment. Notably, we observed a increasing incidence of eschar in STME. Eschar often serves as a key diagnostic clue for clinicians. The high eschar rate in STME cases suggestd that in endemic regions and during high-risk seasons, the presence of eschar should immediately raise suspicion for STME. Additionally, the CSF profile in our STME closely resembled that of viral encephalitis, which can lead to misdiagnosis as viral encephalitis. This finding highlighted the need to consider rickettsial infection in patients with prolonged fever and typical viral encephalitis CSF profiles.

Among our machine learning models, the MLP model demonstrated high predictive accuracy in both the training and test sets. However, the lack of visualization tools in R for MLP models renders it a “black box” model. To address this, we utilized the XGBoost model for enhanced visualization, providing clearer insights into how specific variables influence outcomes. Nonetheless, this study has several limitations. While the SHAP algorithm was utilized to visualize the model, it has notable drawbacks. First, its high computational demand may limit its applicability in rapid clinical decision-making. Second, the interpretability of SHAP is influenced by the complexity of the underlying model, potentially making it difficult for clinicians to intuitively comprehend the results. Additionally, this is a single-center retrospective study, which lacks multi-center validation and may introduce selection bias. Furthermore, the sample size is relatively small. Future studies would benefit from multi-center, large-sample, randomized controlled trials to validate these findings.

## Conclusion

The predictive models based on MLP and XGBoost regression demonstrated strong predictive capability, providing a valuable tool for screening aseptic meningitis in scrub typhus and aiding in the identification of high-risk individuals.

## Authors’ contributions

YL and YW conducted data screening and quality evaluation. YL and YW helped design study. YL and WD wrote the main manuscript text. YL, WD and YW made contributions to the revision of the paper. LY, WD, HB, FJ, YG, TZ, XZ and XY did a lot of data collection work. All authors revised, read and approved the final manuscript.

## Data Availability

The datasets are available from the corresponding author on reasonable request.
